# Bleeding from gastrointestinal angioectasias is not related to bleeding disorders - a case control study

**DOI:** 10.1186/1471-230X-10-113

**Published:** 2010-09-28

**Authors:** Charlotte M Höög, Olle Broström, Tomas L Lindahl, Andreas Hillarp, Gerd Lärfars, Urban Sjöqvist

**Affiliations:** 1 Department of Medicine, Karolinska Institutet, Stockholm Söder Hospital, Stockholm, Sweden; 2 Department of Clinical and Experimental Medicine, Linköping University, Linköping, Sweden; 3 Department of Clinical Chemistry, Malmö University Hospital, Malmö, Sweden

## Abstract

**Background:**

Angioectasias in the gastrointestinal tract can be found in up to 3% of the population. They are typically asymptomatic but may sometimes result in severe bleeding. The reasons for why some patients bleed from their angioectasias are not fully understood but it has been reported that it may be explained by an acquired von Willebrand syndrome (AVWS). This condition has similar laboratory findings to congenital von Willebrand disease with selective loss of large von Willebrand multimers. The aim of this study was to find out if AVWS or any other bleeding disorder was more common in patients with bleeding from angioectasias than in a control group.

**Methods:**

We compared bleeding tests and coagulation parameters, including von Willebrand multimers, from a group of 23 patients with anemia caused by bleeding from angioectasias, with the results from a control group lacking angioectasias.

**Results:**

No significant differences between the two groups were found in coagulation parameters, bleeding time or von Willebrand multimer levels.

**Conclusion:**

These results do not support a need for routine bleeding tests in cases of bleeding from angioectasias and do not show an overall increased risk of AVWS among these patients.

## Background

Angioectasias, also named angiodysplasias in the literature, are vascular malformations that can be found throughout the gastrointestinal tract, with the most common site being the right colon [1,2]. These lesions may occasionally cause severe bleeding but they can also be found in symptom-free patients. In terms of patient presentation, angioectasias are most common in eldery patients undergoing an evaluation for gastrointestinal bleeding. Angioectasias are proposed to be the result of a degenerative process. The prevalence is estimated to 0.9-3.0% in non-bleeding patients and up to 6% in patients with evidence of blood loss [3].

The reason why some patients bleed from their angioectasias and some do not is not yet understood. Chronic renal failure is one mechanism that has been reported in association with bleeding from angioectasias [4].

In 1957 Heyde was the first to report about several patients with aortic stenosis who had massive gastrointestinal bleeding of unknown origin [5]. This publication was followed by larger studies that confirmed this association [6,7]. Selective angiography and subsequent endoscopic techniques allowing direct visualization of the mucosa revealed the appearance of angioectasias as a probable source of bleeding [8]. Thus the connection between aortic stenosis and bleeding from gastrointestinal angioectasias became known as Heyde's syndrome. During the 1980's Heyde's syndrome was questioned in several case-control studies where the association between aortic stenosis and angioectasias could not be reproduced [9,10] but then finally confirmed by Pate et al in a large retrospective study [11]. The link between aortic stenosis and bleeding from angioectasias has been suggested to be an acquired form of von Willebrand disease.

The von Willebrand factor (VWF) molecules are of different sizes, varying from 500 to >20,000 kD. These molecules adhere to platelets when bleeding occurs, causing coagulation. Acquired von Willebrand syndrome (AVWS) is a rare bleeding disorder with many similarities to congenital von Willebrand disease (VWD). There are many causes for AVWS including lympho- or myeloproliferative disorders, solid tumours, auto-antibodies against VWF and cardiovascular disorders [12-14]. Vascular abnormalities, such as aortic stenosis, may cause increased shear rate of the flowing blood that lead to degradation of large VWF multimers in plasma through proteolytic cleavages. The result is an acquired loss of the largest multimers of the von Willebrand complexes [15-17]. These large multimers are important in maintaining hemostasis under the conditions of increased wall shear rates that are also found in angioectasias. This may explain why angioectasias could bleed without an underlying bleeding tendency [8].

When a stenotic aortic valve is surgically removed and replaced with a mechanical valve, previous bleeding from angioectasias stops and coagulation parameters normalize [8,18,19].

Therefore, AVWS may be expected to be found in patients with bleeding angioectasias. Precisely this correlation was observed in a small study by Veyradier et al [20]. In this study eight of nine consecutive patients with bleeding from angioectasias had a loss of the largest multimers of the VWF. When examined with echocardiography seven of eight patients with AVWS also had aortic valve disease.

If the prevalence of AVWS is as high as the study by Veyradier et al suggests, should clinicians perform laboratory tests of VWF in all patients with bleeding from angioectasias? Should these patients be evaluated for aortic valve disease? Should these patients be routinely tested for other bleeding deficiencies?

The aim of this study was to test if unselected patients diagnosed with anemia caused by bleeding from gastrointestinal angioectasias had AVWS or any other bleeding disorder, as determined by abnormal bleeding parameters or loss of coagulation factors. The results of the study group were compared to those from a control group of patients without gastrointestinal angioectasias. Echocardiography was not considered necessary in the case of normal levels of VWF multimers.

## Methods

**Figure 1:**
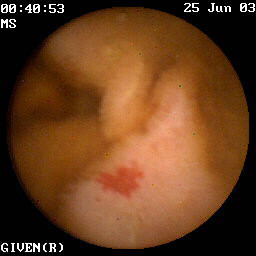
**Angioectasia in the jejunum detected by capsule enteroscopy**. Patient from the study group.

Twenty-five patients with a previous history of gastrointestinal bleeding due to angioectasias in the gastrointestinal tract were identified between 2003 and 2006 using the records of the Department of Gastroenterology at the Stockholm Söder Hospital. These patients were retrospectively enrolled in the study. Two participants were subsequently excluded from the study because of findings suggestive of an on-going malignancy that could have interfered with the bleeding test results. The mean age of the remaining 23 patients was 72 years (35-86). Fifteen of the patients were female (65%). All study participants had gastrointestinal bleeding that was sufficient to cause anemia and 15 participants had been treated with blood transfusions (two units or more). All study participants had undergone upper and lower endoscopy (GIF-160, TL/PCF-160, Olympus Sweden AB, Solna, Sweden) performed by gastroenterologists at our endoscopy unit. Angioectasias were found in the upper gastrointestinal tract in seven patients, in the lower tract in seven patients, in the small intestine in five patients and in multiple locations in four patients. Angioectasias found in the small intestine were diagnosed by means of capsule enteroscopy (PillCam, Given, Yoqneam, Israel). Capsule enteroscopy was performed in patients where no plausible bleeding source was found by means of gastro- and colonoscopy or if bleeding reoccurred after endoscopic treatment of the angioectasias. The diagnosis of angioectasia was based upon endoscope findings of a flat bright reddish lesion, measuring 3-10 mm with a typical appearance (fig 1). The angioectasias were considered to be the only source of bleeding in the study group.

These patients were compared to a retrospective control group consisting of 24 patients diagnosed with diverticulosis, also identified from the records of the Department of Gastroenterology at the Stockholm Söder Hospital spanning the same time period as the study group. The mean age of the control group was 73 (35-86) years and 15 were females (62%). All patients in the control group had a gastroscopy and a colonoscopy without identification of any angioectasias. The indications for endoscopy were gastrointestinal bleeding, abdominal pain or diarrhoea. The control group was of same gender and age composition as the study group which is why matched controls were not considered necessary.

Exclusion criteria for both groups were malignancy, the use of warfarin during the bleeding episode, the absolute need for drugs affecting bleeding parameters, known bleeding disorder, thrombocytopenia, kidney failure (i.e. serum creatinin concentration above 150 mmol/L), alcoholic over-consumption and severe psychiatric illness.

The participants were contacted by phone and were subsequently given written information. Patients enrolled were asked to leave blood samples on one occasion.

All drugs that might affect bleeding parameters were stopped two weeks before blood tests were performed.

The participants were tested for haemoglobin level, platelet count, serum creatinin and activated partial thromboplastin (APT) time. Bleeding time (according to Ivy) was tested utilising a Surgicutt™ device from International Technidyne Corporation (Edison, NJ, USA). For coagulation analysis, blood was collected in Vacutainer tubes containing 1/10 volume sodium citrate, then plasma was separated immediately after blood collection. Plasma was stored at -70°C until analysis. Factor VIII was measured with a chromogenic assay (Chromogenix, Milan, Italy), prothrombin time (PT-INR) with the reagent GHI-131 (Medirox, Nyköping, Sweden), von Willebrand-antigen (VW-ag) and von Willebrand-activity (VW-act) with reagents from Instrumentation Laboratory (Milan, Italy) utilising an ACL Top (IL). All tests were performed at the coagulation laboratory of Linköping University Hospital and were calibrated with manufacturer assigned values through comparison with the WHO reference materials. The laboratory is accreditated according to the standard EN 45001 by the Swedish board for Accreditation and Conformity Assessment (SWEDAC). The results from the external control scheme ECAT were good. The imprecision for vW-act was for the control with mean 1.13 kIE/L C.V. 5.6% (n = 54) and for control with mean 0.29 kIE/L C.V. 7.7% (n = 52), respectively.

**Figure 2:**
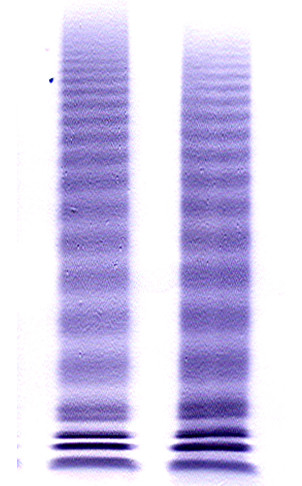
**Western blot shows a slightly depleted level of large von Willebrand multimers in one of the study patients (right) and a patient with normal levels (left)**.

Von Willebrand-multimer (vW-multimer) was analysed with SDS-gelelectrophoresis and von Willebrand-multimer (VW-multimer) with SDS-gelelectrophoresis followed by western blot (fig 2). The multimeric pattern of VWF in plasma was analyzed by electrophoresis in 1,9% SeaKem^® ^HGT(P) agarose gel (Lonza, Rockland, ME, USA) in the presence of sodium dodecyl sulphate followed by immunoblotting with antibodies against VWF as previously described [21,22]. The multimeric distribution was thereafter quantitated using densitometric analysis where peaks 1 through 5 represents small, peaks 6 through 10 intermediate and peaks above 10 large multimers as described by Budde et al[23]. Blood sampling and bleeding-time were performed by one single nurse at our unit during 2006.

Median values with range were used in the text. Non parametric statistics of mean values were used for comparison.

The study was approved by the Ethics Committee in Stockholm (Etikprövningsnämnden, Stockholm, Sweden).

## Results

Hemoglobin was significantly lower in the study group compared to the control group (129 vs 139, p = 0,0294). There was no significant difference in any of the other parameters that were examined. The results are shown in Table 1.

**Table 1:** A comparison of coagulation parameters between 23 patients with bleeding from angioectasias and 24 controls.

Parameter	Study group, mean (min-max)	Control group, mean (min-max)	Significance, P-value < 0,05
Hemoglobin level, g/L	128 (97-151)	141 (113-169)	0,0294

Platelet count, 10^9^/L	262 (140-457)	265 (176-562)	NS

Creatinin, mmol/L	75 (56-136)	76 (62-112)	NS

C-reactive protein, mmol/L	<10 (<10-25)	<10 (<10-20)	NS

PT-INR	1,0 (0,9-1,2)	1,1 (0,9-1,2)	NS

APT-time, sec	35 (28-47)	34 (27-41)	NS

VW-antigen, kIU/L	1,47 (0,68-3,63)	1,50 (0,68-2,48)	NS

VW-activity, kIU/L	1,20 (0,70-3,39)	1,20 (0,69-2,23)	NS

Factor VIII, kIU/L	2,00 (1,01-3,84)	2,0 (0,99-2,52)	NS

Fibrinogen, g/L	3,9 (2,8-5,5)	3,9 (2,9-4,7)	NS

Large VW-multimer, %	28,4 (20,4-32,4)	29,9 (24,1-37,5)	NS

Bleeding time (Ivy), sec	Median* = 270 (130->900)	Median* = 250 (149-570)	NS

* = Median was used instead of mean because the value could not be measured above a certain level

Although the results of the whole study group did not significantly differ from those of the control group, two patients in the study group were found to have slightly depleted levels of the largest multimers of von Willebrand (both 20%, normal level 22-27%), table 2.

**Table 2:** Laboratory values of patients with abnormal high molecular weight VWF or bleeding time (Ivy)

Parameter	Patient 1	Patient 2	Patient 3	Patient 4
Hemoglobin level, g/L	128	125	126	134

Platelet count, 10^9^/L	337	323	150	220

Creatinin, mmol/L	79	70	90	64

C-reactive protein, mmol/L	<10	<10	<10	<10

PT-INR	1,0	0,9	1,1	1,0

APT-time, sec	43	38	33	37

VW-antigen, kIU/L	1,12	2,20	2,23	1,20

VW-activity, kIU/L	0,74	1,43	2,04	1,03

Factor VIII, kIU/L	1,15	2,04	2,12	1,79

Fibrinogen, g/L	5,4	5,5	2,8	3,7

Large VW-multimer, %	20,4	20,6	28	31,4

Bleeding time (Ivy), sec	220	165	>900	>900

Patient 1 was an 82 year old woman with a history of chronic obstructive lung disease and a severe aortic stenosis. She had experienced repeated anemia due to multiple angioectasias in both the duodenum and the caecum. The angioectasias had been treated by means of argon plasma coagulation for several times but the bleeding had reoccurred. All bleeding parameters except the level of large VW-multimer were normal. Currently she needs blood transfusions twice a year.

Patient 2 was a 70 year old woman. She had an acute myocardial infarction in 2002 and was treated with primary coronary intervention. Echocardiography showed an aortic stenosis of medium significance. Anemia was also found. This was due to several angioectasias in the duodenum that was treated repeatedly with argon plasma coagulation. The anemia persisted in spite of treatment and therefore capsule enteroscopy was performed which revealed multiple angioectasias in the jejunum. The angioectasias were treated by means of argon plasma coagulation during peroperative enteroscopy. A subsequent attempt at a second treatment by means of double balloon enteroscopy failed due to strictures in the small intestine. In the last year the patient's haemoglobin level has remained stable without further.

Another two patients in the study group had a prolonged bleeding time (>900s) but no other abnormalities were found in their blood chemistry parameters (table 2, "patient 3 and 4"). Their history did not reveal a general bleeding tendency apart from the fact that they had had bleeding from an angioectesia that stopped after endoscopic treatment by means of argon plasma coagulation. The first patient declined further follow-up but the second patient went through a more thorough investigation of coagulation where the only abnormal finding was a second prolonged bleeding time of 540 s. Neither of them had noticed a general bleeding tendency.

## Discussion

Gastrointestinal angioectasias are most commonly found in elderly people and may in some individuals cause bleeding that can be massive. The reasons for why angioectasias tend to bleed in some cases and not in others are not fully understood. A connection between bleeding from angioectasias and aortic stenosis known as Heyde's syndrome has been a matter of debate for many years. The link between those two diagnoses was found to be acquired von Willebrand syndrome. The aim of this study was to find out if AVWS or any other bleeding disorder was found in higher frequency in patients with a history of bleeding from angioectasias than in a control group. A small study has previously implied a high frequency of AVWS due to cardiovascular disease in a similar study group [20]. It is of clinical importance to know if all patients with bleeding from angioectasias should be routinely tested for bleeding disorders.

In this study, after comparison of bleeding tests and coagulation parameters between study and control groups, no relationship between bleeding from gastrointestinal angioectasias and acquired AVWS or any other bleeding disorder was identified. Only hemoglobin levels differed significantly. If an even larger study group is needed for showing a possible relationship between bleeding from angioectasias and a bleeding disorder, those results would still at the best indicate a very weak relation.

Interestingly there were two patients in the study group who had slightly decreased levels of the largest multimers of von Willebrand. These patients also had known aortic stenosis which is a risk factor for AVWS [8,17,18]. Two other patients had a prolonged bleeding time of more than 900 s. This test though has a low specificity [24] and its diagnostic value is uncertain [25]. At follow up no signs of any general bleeding tendency or bleeding from the intestine after initial treatment of angioectasias were found.

The fact that the blood sampling in most cases was not performed when the bleeding occurred is a limitation of our study. However, since we were seeking a chronic bleeding tendency the time point for blood sampling was not considered a major issue. Another limitation of the study is that a majority of the patients identified with bleeding from angiectasias met one of the exclusion criteria or were unwilling to participate, thus excluding them from enrollement. The median age of the study population was rather high (72 years) reflecting that angioectasias are of degenerative nature [3] and bleeding from those are reported to be common among elder patients with aortic stenosis [26]. High age is also a risk factor for comorbidity leading to exclusion from the study. Screening for aortic valve disease with echocardiography was not included in the study design, because our intention was not to confirm an association between AVWS and aortic stenosis. The two patients from the study group with depleted levels of large VW-multimers had known aortic stenosis already confirmed by echocardiography before the onset of the study.

The question of why some patients tend to bleed from their angioectasias is neither fully answered by findings of AVWS nor by any other coagulation disorder tested for in this study. Chronic renal failure was one of the exclusion criterions but might otherwise be a factor of some importance. High age is another factor that can be supposed to increase the risk. These results may reflect that bleeding from angioectasias is caused by multiple factors where some of them may still be unknown.

## Conclusions

Based on the observations in this study we suggest that there is no need for routine testing for acquired von Willebrand disease or other bleeding disorders in patients with bleeding from gastrointestinal angioectasias. In cases of known aortic stenosis or repeated bleeding from the angioectasias, testing of VWF multimers might be considered.

## Competing interests

The authors declare that they have no competing interests.

## Authors' contributions

CH participated in conception and design of the study, recruiting patients, collecting data and drafting of the manuscript. OB, US, and GL participated in conception and design of the study, discussions and drafting of the manuscript. TL and AH performed laboratory analysis and contributed with important expert knowledge and discussions. All authors read and approved the final manuscript.

## Pre-publication history

The pre-publication history for this paper can be accessed here:

http://www.biomedcentral.com/1471-230X/10/113/prepub
